# Pathological Similarities in the Development of Papillomavirus-Associated Cancer in Humans, Dogs, and Cats

**DOI:** 10.3390/ani12182390

**Published:** 2022-09-13

**Authors:** Alfredo Cruz-Gregorio, Ana Karina Aranda-Rivera, José Pedraza-Chaverri

**Affiliations:** Laboratory F-315, Department of Biology, Faculty of Chemistry, National Autonomous University of Mexico, Mexico City 04510, Mexico

**Keywords:** squamous cell carcinoma, *Canis familiaris* papillomavirus, *Felis catus* papillomavirus, human papillomavirus, pathological similarities in cancer, E5, E6, and E7 oncoproteins

## Abstract

**Simple Summary:**

Papillomavirus (PV) infection affects many species, including humans and domestic animals, such as dogs and cats. Some of these infections involve the development of cancer due to the presence of PV. There are similarities in the pathology of these three PV-associated cancers, which may provide crucial insights into cancer development in these species, extrapolating both markers and possible treatment in the three species. For example, the oncoproteins E5, E6, and E7 are the main causes of the development of cancer associated with PV, and the possible therapies associated with the blockage or reduction of these oncoproteins can be of great benefit for the reduction and/or elimination of cancer associated with PV. Thus, our review focuses on the similarities in the context of pathology and biomarkers in canine, feline, and human cancers associated with PV. We review the main biomarkers, E5, E6, and E7 oncoproteins, and their overexpression in *Canis familiaris*, *Felis catus*, and human papillomavirus and their association with the development of cancer. Furthermore, we also discuss that a potential treatment for PV-related cancer is the reduction or blocking of these oncoproteins.

**Abstract:**

*Canis familiaris*, *Felis catus*, and human papillomavirus are nonenveloped viruses that share similarities in the initiation and development of cancer. For instance, the three species overexpress the oncoproteins E6 and E7, and *Canis familiaris* and human papillomavirus overexpress the E5 oncoprotein. These similarities in the pathophysiology of cancer among the three species are beneficial for treating cancer in dogs, cats, and humans. To our knowledge, this topic has not been reviewed so far. This review focuses on the information on cancer research in cats and dogs comparable to that being conducted in humans in the context of comparative pathology and biomarkers in canine, feline, and human cancer. We also focus on the possible benefit of treatment associated with the E5, E6, and E7 oncoproteins for cancer in dogs, cats, and humans.

## 1. Introduction

*Canis familiaris* (Cf), *Felis catus* (Fc), and human (H) papillomavirus (PV) are nonenveloped viruses with a capsid that envelops the genome of a single double-stranded deoxyribonucleic acid (dsDNA) molecule [1]. Both CfPV and HPV genomes are organized in three regions: (1) the long control region (LCR) that modulates viral replication and viral transcription, (2) the early region composed of six open reading frames (ORFs): E1, E2, E4, E5, E6, and E7, and (3) the late region formed by the ORFs of L1 and L2 that code for the proteins E1, E2, E4–E7, L1, and L2, respectively [2]. Unlike CfPV and HPV, the FcPC genome does not express the E4 and E5 proteins [3]. The E1 protein is a helicase implicated in viral replication, which needs E2 binding to increase E1 specificity to its DNA sequence target [2]. Importantly, the E2 protein also downregulates the viral transcription of E6 and E7 oncoproteins [4]. The release of virions by disrupting the cellular network of cytokeratin filaments is induced by the E4 protein [5]. E5, E6, and E7 are the oncoproteins that target different essential cellular proteins to induce cell immortalization and proliferation [6].

CfPV, FcPV, and HPV share similarities in the initiation and development of cancer. However, to our knowledge, this topic has not been reviewed so far. This review focuses on the similarities in the pathophysiology of cancer among the three species that are beneficial for treating cancer in dogs, cats, and humans. Since the HPV is the main papillomavirus studied, we first mention the HPV genome and HPV viral life cycle. Then, we describe the HPV oncoproteins E5, E6, and E7 and similarities in the pathophysiology of cancer among the three species, Cf, Fc, and humans, focusing on the possible benefit of treatment associated with the latter oncoproteins for cancer in dogs, cats, and humans.

## 2. HPV Viral Life Cycle

**Figure 1:**
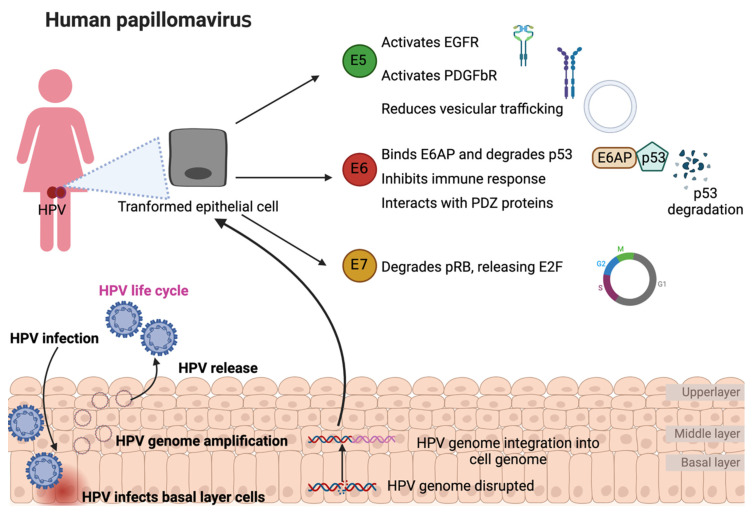
Human papillomavirus (HPV) infection, life cycle, and oncoprotein overexpression and functions. HPV infects the basal layer cells, amplifies its HPV genome, and HPV virions releases are induced to complete its life cycle. The disruption of the HPV genome might cause its integration into the infected cellular genome. The latter induces the overexpression of early (E) oncoproteins, E5, E6, and E7, inducing the transformation of infected cells. EGFR: epidermal growth factor receptor; PDGFbR: platelet-derived growth factor b-receptor; E6AP: E6-associated protein; PDZ: postsynaptic density protein 95 (PSD95)/discs-large (Dlg)/zonula occludens-1 (ZO-1); pRB: retinoblastoma protein; E2F: E2 transcription factor. The figures were originally created in BioRender.

HPV reaches and infects the cells of the basal layer of the squamous epithelium through wounds in the epithelial layer (Figure 1) [7]. For this, the HPV L1 protein must bind with heparan sulfate proteoglycan receptors, which induces conformational changes of the viral capsid to permit the transference of the viral particle to a second receptor, and the L2 protein interacts with the S100A10 subunit of the annexin A2 heterotetramer in the cell membrane [8,9]. The latter promotes HPV internalization into the host cell by endocytosis, which converges in HPV transportation through an early endosome and late endosome, where the L1 protein dissociates from L2, inducing the disassembling of viral capsid during its transport to the Golgi network. However, L1 is not entirely dissociated from L2–viral DNA during this process because this interaction is necessary for enhancing virus transportation into the nucleus [10]. It has been shown that L1 protects the viral DNA from degradation and allows the formation of the L2–viral DNA complex. Then, the L1–L2–viral DNA complex is transported through the trans-Golgi network and, afterward, into the infected cell nucleus [11]. In the nucleus, the L1–L2–viral DNA complex is delivered where the transcription and replication of HPV are carried out [12]. Note that the replication of the HPV genome requires the differentiation of basal epithelial cells since undifferentiated basal epithelial cells do not express the cellular transcription factors (TFs) such as specificity protein 1 (SP1) and transcription factor IID (TFIID) to induce HPV early protein expression [13]. Consequently, viral genome amplification is maintained at a low copy number along with low expression levels of E1, E2, E5, E6, and E7 [12]. In the middle layer, as the epithelium is differentiated, the TFs that induce viral genome amplification are expressed, inducing HPV early proteins to increase their expression. Thus, the levels of HPV E6 and E7 oncoproteins increase, and the interactions of these oncoproteins with p53 and pRB, respectively, are induced. The latter induce the degradation of p53 and pRB, preventing cell cycle arrest and cell death via apoptosis but provoking cell cycle activation to progress from G1 to the S phase [14,15]. Moreover, the E1 and E2 proteins augment their expression in the differentiated epithelium, increasing the replication of the HPV genome in high quantities. E4 induces cell keratin disruption in the upper layer, allowing a virion release after the L1 and L2 proteins form the HPV capsid to house its genome [12].

On rare occasions, the HPV life cycle can be interrupted by the integration of the virus genome into the cellular genome, a negative event to produce virions; therefore, it is not within the virus’s life cycle (Figure 1). Integration has been considered a necessary event for progression to cervical cancer, as the HPV genome has been reported to be episomally present in low-grade lesions, whereas viral genome integration is observed in the early stages of advanced invasive carcinoma [16]. HPV genomic integration into the cell genome results in an increased expression and stability of the transcripts encoding the viral oncoproteins E6 and E7. The latter is because there is a break or deletion of the HPV E2 protein open reading frame (ORF). Since E2 is a protein that downregulates both E6 and E7, the loss of the E2 ORF results in an increased expression of the E6 and E7 oncoproteins [17]. It is important to point out that there is a low percentage of cervical cancer where the HPV genome is episomally maintained. Even so, the development of cancer associated with these cases has also been related to an increase in E6 and E7 oncoproteins as a consequence of an increase in the episomal form of the virus or the epigenetic modification that induces E2 silencing (Figure 1) [18,19]. The increase in copies of the virus achieves, although less efficiently, the same effect as the integration of HPV [20]. Since there is an overexpression of PV oncoproteins in both dogs and cats, it is expected that the same mechanisms of cancer induction are present in these species. One of the mechanisms that have been suggested to induce the integration of the viral genome into the cellular genome is the presence of oxidative stress (OS) [21,22]. OS induces DNA damage, facilitating hybridization between the two genomes, the cellular and the viral, through the DNA damage repair (DDR) machine [23]. Although the above mechanisms have only been studied in HPV-associated cancers, it is very likely that these same mechanisms are present in PV-associated cancers in dogs and cats.

## 3. Pathophysiology in Human, *Canis familiaris* (Cf), and *Felis catus* (Fc) Papillomavirus-Associated Cancer Development

High-risk HPV (HR-HPV) infection is recognized as a requisite for cervical cancer development [17,24]. Furthermore, HR-HPV has been recognized as the causative agent for other anogenital cancers such as anus or vagina and a subset of head and neck cancers [25,26]. It has been shown that in humans, HPV types 16 and 18 are the most prevalent causative agents of HPV-related cancer. CfPV and FcPV have also been found to be associated with squamous cell carcinomas (SCC). In dogs, CfPV2 leads to metastatic SCC in immunosuppressed dogs [27], and CfPV3 has been detected in a malignant canine epidermodysplasia verruciformis (EV) [28]. Moreover, CfPV7 was also detected in cutaneous SCCs in dogs [29]. Regarding FcPV, FcPV2 to 6 are associated with SCC [3]. Interestingly, all three species are similar in the molecular mechanism for cancer induction, that is, the induction and overexpression of the oncoproteins E5, E6, and E7 for CfPV and HPV and E6 and E7 overexpression for FcPV, which are described in later sections. Note that in the three species, the immune response induces PV clearance; however, immunosuppressed species are prone to developing SCC [30]. Thus, for example, the predisposition to SCC in immunosuppressed people is common in people with EV [31]. This predisposition is also similar in cats and dogs, where cats and dogs suffering from EV in a state of immunosuppression lead to the development of SCC [30]. In humans, the genetic defect is localized in epidermodysplasia verruciformis (EVER) 1 and EVER2 genes that encode for transmembrane channel-like (TMC) proteins 6 and 8 [32]. The progression from EV to SCC in humans provides strong evidence of a possible similar mechanism in dogs and cats.

## 4. PV E6 Oncoprotein as a Biomarker of Carcinogenesis in HPV, CfPV, and FcPV

Although E6 and E7 oncoproteins collaborate in the cell transformation process, both have distinct biological activities contributing to malignancy development. For instance, E6 perturbs many different cell biological activities in the cell [33]. One of the main biological functions of E6 is the inhibition of apoptotic cell death. E6 can induce epithelial-mesenchymal transition and perturb aspects of the immune response pathways. E6 also plays a crucial role in cell immortalization, activating the enzyme telomerase [33]. The latter processes are because the E6 protein targets and associates with many different cellular proteins. These interactions occur between E6 and proteins harboring LxxLL motifs or PDZ domains [34]. The ubiquitin ligase E6-associated protein (E6AP) is one partner that bears this motif. E6AP is a crucial protein in the degradation of p53 tumor suppressor via the proteasome; consequently, E6AP is part of E6’s ability to induce cancer [34]. Note that p53 is not mutated in HPV-related cancers, and the ability of E6 to induce its degradation is thought to be analogous to mutational inactivation in other cancers [35]. Interestingly, a loss of association of E6 with E6AP causes a rapid degradation of E6 through the proteasome, indicating the stability and increased lifespan of E6 induced by the E6–E6AP complex [36]. Therefore, designing small molecules that inhibit the association between E6 and E6AP could provide unique opportunities for potential therapies for PV-related cancers. In fact, various lead compounds and peptides have been developed as inhibitors of the interaction between E6 and E6AP, avoiding p53 degradation induced by E6 [37,38]. However, it is still necessary to verify that these compounds are effective in vivo.

E6 also interacts with proteins containing postsynaptic density protein 95 (PSD95)/discs-large (Dlg)/zonula occludens-1 (ZO-1), known as PDZ domains [39] (Figure 1). PDZ domains are found in proteins involved in cell signaling pathways and proteins present at sites of cell-cell contact [40]. Unlike the interactions of LxxLL with E6 that occur within the entire HPV spectrum (both low- and high-risk), PDZ domain recognition and binding only occur in high-risk HPV types [41]. This feature alone could contribute to the development of malignancy. The latter is because proteins such as discs large (Dlg) and scribble (Scrib) have PDZ domains. Both proteins, Dlg and Scrib, have been classified as tumor suppressors linked to the regulation of cell polarity [42]. Indeed, tumor-associated viruses have been shown to regulate cell polarity as a common mechanism in developing malignancy [43].

Interestingly, it has been shown that the inhibition of the interaction of E6 with the proteins of the PDZ domains could have marked beneficial effects for the elimination of the virus since viral amplification and, therefore, its replication is prevented [44]. Blocking the interaction of E6–PDZ domain proteins prevents the ability of E6 to induce cell transformation in different models, both in vivo and in vitro [45]. Therefore, by blocking the ability of E6 to interact with proteins harboring PDZ domains, the replication capacity of the virus and its transforming activity would be diminished, which would also prevent cooperation with E7 in the last stages of malignant progression [46].

In summary, targeting the interaction between LxxLL and E6 offers a potential therapeutic intervention against E6 in PV-induced cancer, taking advantage of the fact that the LxxLL–E6 interaction is conserved across multiple PV types and thus can be applied in therapies against the expression of PV E6 that binds to the LxxLL motifs. The latter does not happen with the E6–PDZ interaction because the possible blockers of this interaction are only for HR-HPV and cannot be used for LR-HPV. In addition, the high degree of variation between different HPV types and the post-translational modification to which the E6 protein is subjected could be undermined. The inhibition of LxxLL interactions, compared to PDZ interactions, might have a more significant potential to block E6 function, both in terms of blocking different PV types and high E6 substrate selection. However, the suppression of E6 function using both interaction blocks would offer the best prospects for developing therapies to treat PV-induced cancer. It is important to note that these therapies would be more effective in treating late-stage cancer than early-stage cancer, as E6 has a more significant effect on late-stage than early-stage malignancy.

Several similarities have been reported between HPVE6 and CfPVE6. For instance, CfPV E6 contains the PDZ domain protein binding motif, suggesting that E6 might bind to PDZ proteins like HPV16 E6, which is highly related to cellular transformation [27]. Moreover, a study reported that E6 affected immunodeficient dogs because E6 interfered with the expression of antivirals cytokines such as interferon (IFN)-β and the IFN-stimulated gene interferon-induced protein with tetratricopeptide repeats (IFIT)1 in keratinocytes [47]. The latter avoided the immune response activation, which impeded virus elimination. Although the authors did not investigate the PDZ profile in this work, it is known that the virus evades immune system by targeting the PDZ proteins of the immune system [48].

The oncogenicity of human E6 is associated with proliferation and apoptosis evasion associated with p53 degradation via the proteasome. However, the oncogenic mechanism of CfPV2 E6 might not be related to the well-described ability of E6 to degrade p53. Quinlan et al. [49] reported that keratinocytes that expressed CfPV2 E6 exposed to UV did not decrease p53 levels nor avoid the expression of p21, B cell lymphoma (BCL)2 antagonist/killer (Bak), and Bcl-2-associated X (Bax). These results suggest that other p53-independent oncogenic undetermined mechanisms might be present in the malignity of E6 in dogs.

**Figure 2:**
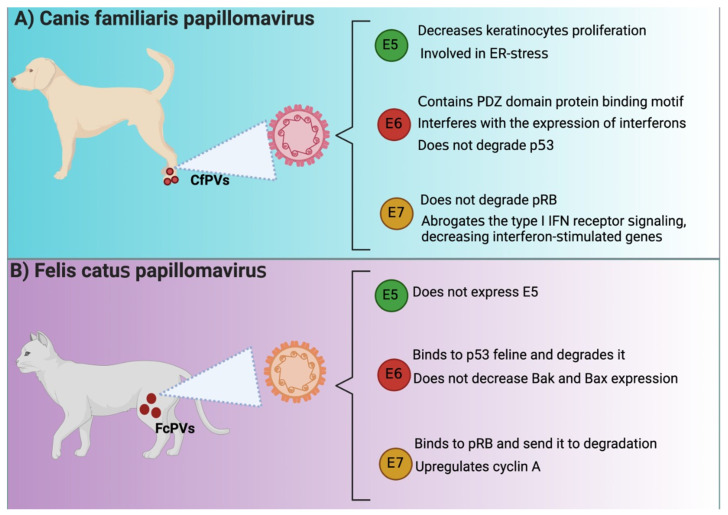
E5, E6, and E7 in *Canis familiaris* papillomavirus (CfPVs) and *Felis catus* papillomavirus (FcPVs). Similarities and differences among E5, E6, and E7 oncoproteins from HPV and (**A**) *Canis familiaris* papillomavirus (CfPVs) and (**B**) *Felis catus* papillomavirus (FcPVs). IFN: interferon; ER: endoplasmic reticulum; pRB: retinoblastoma protein; PDZ: postsynaptic density protein 95 (PSD95)/discs-large (Dlg)/zonula occludens-1 (ZO-1); Bak: B cell lymphoma (Bcl) 2 antagonist/killer; Bax: Bcl2-associated X. The figures were originally created in BioRender.

Like HPV16 E6, FcPV2 E6 binds and degrades p53, inducing proliferation and avoiding apoptosis. Both E6 and E7 avoid p53 and p21 accumulation after ultraviolet B (UVB) exposition and the accumulation of apoptotic factors [50], suggesting an impairment in apoptosis induction. Interestingly, E6 and E7 did not decrease Bak and Bax, suggesting that these proteins are not a target of FcPV oncoproteins, as previously demonstrated in the case of HPV E6 and E7. In this sense, apoptosis evasion might be associated with other mechanisms independent or dependent on p53 [50]. Thus, it has been described that the FcPV2 E6 protein has some similar binding sites to the HPV-16 E6 protein, which could explain the oncogenic activity of FcPV2 (Figure 2).

## 5. PV E7 Oncoprotein as a Biomarker of Carcinogenesis in HPV, CfPV, and FcPV

The E7 oncoprotein has a high capacity for transformation due to its ability to induce the degradation of the tumor suppressor protein pRB, via the proteasome [51]. pRB is a critical protein in the control of cell proliferation, and the degradation of pRB induces the release of E2F, inducing cells to enter the synthesis phase (S phase) of the cell cycle [52]. E7 is also involved in other cellular processes such as gene transcription, epigenetic reprogramming, protein degradation, genomic integrity, cell death, and metabolism [53]. HPV E7 is a phosphoprotein with three conserved regions 1/2/3 (CR1/2/3). The CR3 region at the carboxyl-terminal contains a zinc finger domain with two CXXC motifs separated by 29 amino acid (aa) residues. This region is responsible for mediating the E7 interaction with cellular proteins such as pRB [54]. To our knowledge, there are no studies testing blockers of the interaction between the CXXC binding motifs of E7 and their target proteins, such as pRB, which need to be studied for potential possibilities to reduce carcinogenesis in the three E7-associated species.

FcPV has been associated with the downregulation of pRB during SCC, similar to HPV-associated cancers (Figure 2) [50]. In contrast, it is essential to mention that in the case of CfPV, E7 lacks Rb binding site, suggesting that it cannot degrade retinoblastoma [27]. The lack of binding of E7 to Rb has also been related to a substitution of serine substituted for cysteine in the LXCXE motif, implying that other mechanisms might be involved in the lack of regulation of Rb by E7 [55]. Thus, these data suggest that CfPV E7 uses an alternative mechanism to induce cell cycle activation since CfPV E7 is transcriptionally active in vivo [50]. Furthermore, Quinlan et al. [47] showed that CfPV2 E7 avoids immune response activation because it abrogates the IFN type I receptor signaling, decreasing interferon-stimulated genes and immune response [47]. In the case of FcPV2, it has been found that both FcPV2 E6 and E7 can downregulate molecular targets p53 and pRB [50], implicated in carcinogenic processes such as HR-HPV-related cancer. Unlike CfPV E7, FcPV E7 binds to Rb, inducing its degradation, leading to the transcription of the target genes cdc2 and cyclin A. [56]. In line with the latter, it has been shown that the transfection with E7 in Crandell Rees feline kidney (CRFK) epithelial cells showed an upregulation of cdc2 and cyclin A [50]. Thus, although CfPV E7 does not induce Rb degradation, FcPV E7 does so in a similar manner to HPV. It should be noted that the independent study of cell cycle activation by CfPV E7 on Rb degradation could yield new insights into E7-independent cell cycle activation by HPV E7.

## 6. PV E5 Oncoprotein as a Biomarker of Carcinogenesis in HPV, CfPV, and FcPV

E5 is the smallest protein encoded by HPV and CfPV, with only 83 aa and 43 aa, respectively. E5 is located mainly in the ER and the Golgi apparatus for two species [57]. E5 has been identified as an oncoprotein since it induces cell proliferation associated with overexpression of the epidermal growth factor receptor (EGFR) [58]. Likewise, it has been shown that HPV E5 binds to the 16K subunit of the vacuolar V-ATPase of the endosomes, decreasing its activity and inhibiting its acidification, which results in reducing vesicular trafficking [59]. On the other hand, it has been shown that in primary keratinocytes harboring HPV E5, the levels of cyclooxygenase 2 (COX 2) decrease, impacting the reduction of ER stress responses [60]. Moreover, the presence of E5 stimulates the proteasome-mediated degradation of Bax, a proapoptotic protein that downregulates cell death via apoptosis [61]. Because E5 is the least studied oncoprotein of the three oncoproteins E5, E6, and E7, studies of the motifs for the interaction with its target proteins are not common, indicating an urgency in these studies, which will allow a better use for the reduction of the oncogenic properties of this oncoprotein.

E5 from CfPV is also localized in the endoplasmic reticulum (ER), like E5 from HPV, and similar to HPV E5, CPV E5 decreases keratinocytes proliferation and induces cell cycle arrest in the G1 phase [62]. However, the coexpression of E6/E7 with E5 decreased the effects caused by E5; that is, the three oncoproteins induce the proliferation of keratinocytes. It has been shown that this effect on proliferation is due in part to the fact that the presence of CfPV E5 in the ER induces ER stress and that the coexpression of E6/E7 minimizes this effect by inducing cell proliferation [62]. The mechanism in E5-induced ER stress involves the processing of XBP1 by increasing XPBP1 mRNA sliced form. XPBP1 acts as a transcriptional activator promoting the expression of genes related to unfolded protein response (UPR), which activates even more ER stress [62]. Note that ER stress is associated with the induction of apoptosis to trigger cell death by activating caspase-12 and then caspase 3 [63]. Thus, E6 and E7 might avoid apoptosis by inhibiting E5-induced ER stress.

In summary, the similarity in HPV oncoprotein expression and status in developed cancer could provide critical information on the action of their viral counterparts in both dogs and cats, as well as on host cell physiology, when studying these oncoproteins in humans. The same happens with the study of PV in cats and dogs, which can be extrapolated to studies of PV in humans, so it is necessary to study these two former species to benefit the three species. For example, it has been reported that Fc mammary carcinoma cancer may be useful as a model for human breast cancer because the markers and pathology are very similar to human breast cancer [64,65,66]. These similarities could occur with PV-associated cancers in humans, cats, and even dogs.

## 7. Conclusions and Conclusions Remarks

Malignant lesions associated with HPV, CfPV, and FcPV2 make PV of particular concern, especially since in some populations of domestic dogs and cats, and even in humans, PVs are highly prevalent. The similarity in the expression and status of HPV, CfPV and FcPV oncoproteins associated with cancer development could provide advantageous information on the decreased oncogenic potential of these PVs. Furthermore, the study of these biomarkers could be extrapolated between the three species, and oncoprotein blockade therapies could be used for all three cases since, as mentioned, the binding of E6 with LxxLL binding motifs in essential proteins for carcinogenesis, such as AP-1, is too robust to be able to treat with all three species.

## Data Availability

Data is openly available in a public repository that issues datasets with https://pubmed.ncbi.nlm.nih.gov/ (accessed on 17 August 2022).
